# Motile Cilia in Female and Male Reproductive Tracts and Fertility

**DOI:** 10.3390/cells13231974

**Published:** 2024-11-28

**Authors:** Dorota Wloga, Ewa Joachimiak, Anna Osinka, Salman Ahmadi, Sumita Majhi

**Affiliations:** Laboratory of Cytoskeleton and Cilia Biology, Nencki Institute of Experimental Biology of Polish Academy of Sciences, 3 Pasteur Street, 02-093 Warsaw, Poland; e.joachimiak@nencki.edu.pl (E.J.); a.osinka@nencki.edu.pl (A.O.); s.ahmadi@nencki.edu.pl (S.A.); s.majhi@nencki.edu.pl (S.M.)

**Keywords:** cilia, flagella, fallopian tube, uterus, endometrium, efferent ductules, infertility, ectopic pregnancy

## Abstract

Motile cilia are evolutionarily conserved organelles. In humans, multiciliated cells (MCCs), assembling several hundred motile cilia on their apical surface, are components of the monolayer epithelia lining lower and upper airways, brain ventricles, and parts of the reproductive tracts, the fallopian tube and uterus in females, and efferent ductules in males. The coordinated beating of cilia generates a force that enables a shift of the tubular fluid, particles, or cells along the surface of the ciliated epithelia. Uncoordinated or altered cilia motion or cilia immotility may result in subfertility or even infertility. Here, we summarize the current knowledge regarding the localization and function of MCCs in the human reproductive tracts, discuss how cilia and cilia beating-generated fluid flow directly and indirectly contribute to the processes in these organs, and how lack or improper functioning of cilia influence human fertility.

## 1. Introduction—Cilia, the Evolutionarily Conserved Structures

Cilia and homologous structures, flagella, are evolutionarily conserved, microtubule-based, eukaryotic cell protrusions. In modern unicellular and multicellular organisms belonging to diverse evolutionary lineages, cilia and flagella show a high level of conservation at the proteomic and ultrastructural levels. This finding strongly suggests that cilia-like organelles were formed by the last eukaryotic common ancestor (LECA) before it diverged into eukaryotic lineages [1,2,3,4,5,6].

Based on their function, cilia are generally divided into two categories: (i) sensory and (ii) motile cilia. The sensory cilia (including primary cilia) are most often solitary structures. They receive environmental signals via ciliary membrane-docked receptors and transmit them to the cell body [7]. The so-called motile cilia and flagella enable a shift of the secreted substances along the surface of the ciliated cell and cell movement, but they can also perform sensory functions [8,9,10,11]. A motile cilium/flagellum can be assembled as a single organelle per cell (e.g., in some protists, nodal cilia in vertebrates, sperm cells [12,13,14] or multiple structures: (i) as a pair (e.g., *Chlamydomonas* [15]), or (ii) as several (e.g., *Giardia* [16]) to (iii) several hundred organelles (e.g., ciliates, multiciliated cells (MCC) [17]). Not only the number but also the length of motile cilia and flagella can vary depending upon the organism/cell type. The length of motile cilia usually varies between 5–10 µm, while flagella are longer structures, being, in human sperm cells, approximately 60 µm long [18] but reaching even a few centimeters in the sperm cells of *Drosophila bifurca* [19,20].

Cilia assemble from basal bodies, the centriole-homologous structures, with a skeleton composed of nine microtubular triplets (9 × 3 + 0). The most outer microtubule of each basal body triplet is terminated at the level of the basal body’s distal end. The transition of the basal body microtubular triplet to the ciliary outer doublets is accompanied by gradual changes in the circumferential arrangement of the microtubular doublet [21].

The origin of the basal body(-ies) in a cell assembling primary cilium and in a multiciliated cell is different. In the case of the primary cilium-forming cell, the older of the two centrioles of the centrosome, the mother centriole, undergoes transformation, migrates, and anchors to the cell surface to become a basal body (in cycling cells, the primary cilium is disassembled and the mother centriole released from the cell surface before mitosis) [22]. In an animal cell determined to assemble multiple motile cilia, multiple new centrioles (future basal bodies) are nucleated near the parental centrioles and (most of them) in the vicinity of the deuterosome [17].

Basal bodies have accessory structures: transition fibers positioned at the distal end corresponding to the centriole distal appendages, basal foot/feet (likely corresponding to the subdistal appendages), and one or more ciliary rootlet(s) extending from the basal body’s proximal end into the cell interior. The accessory structures anchor the basal body to the cell surface and connect to the cellular microtubule and actin network. The basal body of the primary cilium assembles multiple basal feet, and their number depends upon the cell type. In contrast, the basal body of the motile cilium has one basal foot, extending in the direction of the ciliary effective stroke (the direction of the fluid flow). The architecture and protein composition of the basal foot in the MCCs partly differ from those of basal feet present in cells assembling a primary cilium [23,24,25,26,27,28,29,30,31,32].

A short compartment between the distal end of the basal body and the proximal end of the cilium, the transition zone, has a unique ultrastructural organization and serves as a gate, controlling transport to and from the ciliary shaft [33].

In contrast to other microtubule-containing cell protrusions, the ciliary microtubular skeleton, the axoneme, is a highly stable and uniquely organized structure. It is composed of nine microtubular doublets, positioned at the cilium periphery (of note, in some organisms, the number of doublets can be different [34]). The peripheral doublets, also called the outer doublets, are extensions of two out of three microtubules of each basal body microtubular triplet. The outer doublet is composed of a 13-protofilament A-tubule (protofilaments A1-A13) and a 10-protofilament B-tubule (protofilaments B1-B10) that is firmly connected to the A-tubule wall, either directly (tubulin–tubulin interactions supported by other proteins, so-called outer junction [35]) or indirectly, via non-tubulin proteins forming a protofilament-like structure (B11), so-called inner junction [36,37,38,39,40].

**Figure 1:**
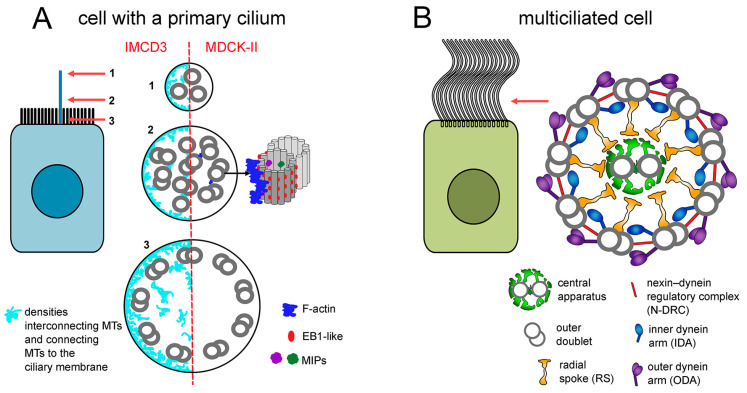
Schematic representation of the ciliary shaft. The ultrastructure (cross-sectional views) of a typical (**A**) non-motile primary cilium with 9 × 2 + 0 microtubule organization and (**B**) motile cilium with 9 × 2 + 2 microtubule arrangement and major ciliary complexes. The red arrow indicates the level at which the cilium cross-section is depicted. In the primary cilium, the canonical 9 × 2 + 0 microtubule organization is only visible at the cilium base (cross-section 3, arrow 3). More distally, the outer doublets lose their symmetry and shift into the cilium center; additionally, one by one, the B-tubules are terminated, and the cilium diameter is reduced (cross-section 2, arrow 2). At the cilium tip (cross-section 1, arrow 1) all B-tubules and most of the A-tubules are terminated, and thus only a few singlets are visible. Note the presence of (i) network-like densities (cyan) along the whole primary cilium length, interconnecting microtubules and microtubules to a ciliary membrane (IMCD3 cell, based on images presented by [42]) or (ii) proteins (MIPs) in the A-tubule lumen, EB1-like protein between protofilaments except in the tubule seam, and F-actin (MDCK-II cell, based on [43]). For the ultrastructural organization of the entire motile cilium, transition zone, and basal body, please see [54], Figure 1.

The presence of nine microtubule outer doublets is a characteristic feature of eukaryotic cilia/flagella (Figure 1). In primary cilia, the 9 × 2 + 0 pattern (nine doublets without additional microtubules) is maintained only in the basal part of the cilium. While moving toward the cilium’s distal end, the microtubule arrangement changes and becomes more bundle-like. Such a change is associated with (i) the reduction of the number of doublets, (ii) the gradual appearance of the microtubule singlets due to the B-tubule termination (rarely, the A-tubule termination), (iii) the inward-shifting and twisting of the remaining microtubules, and (iv) diminished cilium diameter [41,42,43]. In IMCD3 cells, the primary cilia microtubules are interconnected and also connected to the ciliary membrane by the densities of as-yet unknown protein composition [42]. In MDCK-II cells, the ciliary microtubules are accompanied by the luminal proteins (MIPs), F-actin, and EB1-like protein [43], while in RPE1 primary cilia, by CCDC66, a MAP protein [44], (for review [45,46,47]).

In motile cilia, flagella, and some sensory cilia [7,48], the axoneme structure is more complex. In motile cilia, the basic 9 × 2 microtubular skeleton is most frequently accompanied by two centrally positioned single microtubules and thus has a 9 × 2 + 2 organization. However, some motile cilia, such as the majority of nodal cilia in mouse embryos and approximately half of the cilia in the rabbit node, lack central microtubules (the remaining nodal cilia have two or, in rabbits, even four central microtubules) [49,50]. Furthermore, in some insects’ sperm flagella, there is an additional set of nine single microtubules, so-called accessory tubules, which are positioned between the outer doublets and flagellar membrane and are composed of a various number of protofilaments [34,51,52,53].

In motile cilia and flagella, both central and outer doublet microtubules serve as docking sites for multiprotein complexes, which are periodically distributed along microtubules and whose activity generates and regulates cilia/flagella motions. Moreover, a significant number of proteins interact directly with either the luminal microtubule surface (so-called MIPs, microtubule inner proteins) [35,55,56,57,58,59] or outer microtubule surface, forming a network which can mediate and/or coordinate the interactions between ciliary complexes or determine their position (e.g., ciliary ruler; see below) (for review [54]).

The motor protein-containing outer (ODA) and inner (IDA) dynein arms, radial spokes (RSs), and nexin–dynein regulatory complex (N-DRC) are the main complexes of outer doublet microtubules. These large, multiprotein structures can be detected using TEM (transmission electron microscopy), and their arrangement along the microtubule doublet repeats every 96 nm, marking so-called axonemal/ciliary units [54,60]. Each ciliary unit comprises (i) four ODAs, identical in protein composition and function, containing (depending on the species) two or three dynein heavy chains, (ii) seven IDAs—one two-headed (containing two dynein heavy chains), IDAf/I1, and six single-headed, IDAa-e and IDAg, which partly differ in their protein composition and function, (iii) three structurally and biochemically non-identical RSs, and (iv) a single N-DRC, believed to coordinate and regulate the activity of ciliary complexes within the 96-nm unit and connecting the adjacent outer doublets [54,61]. Recent analyses based on combined genetic, biochemical, cryo-EM, and molecular modeling approaches brought significant progress, not only in the deciphering of the protein composition of those large ciliary complexes but also in enabling the identification of small ciliary complexes that likely play a role as ciliary regulators or connectors. These are: (i) MIA complex, composed of CFAP73/CCDC42B and CFAP100/CCDC37, positioned between N-DRC and IDAf/I1 [62], (ii) CCDC113/CCDC96 complex, connecting N-DRC and IDAg [63] and recently shown to also contain another protein, CFAP337 [64], (iii) tether/tetherhead complex (T/TH), composed of CFAP43 and CFAP44 [65,66], (iv) CCDC39 and CCDC40, forming a ciliary ruler [67], (v) filament-forming proteins, e.g., CFAP57 [68], and others, including CCDC146 and CCDC147 [69].

The so-called projections are multiprotein complexes of the central microtubules, named C1a-f and C2a-e. Together with central microtubules and a connecting bridge-like structure, they form the central apparatus [70].

The above-described ciliary complexes are distributed along the axoneme except for the cilium base, the so-called transition zone, and the very distal part of the cilium, named the ciliary tip, both having specific ultrastructure and protein composition [29,33,71].

It is estimated that motile cilia/flagella are composed of several hundred proteins. In the last decade, enormous progress has been made in deciphering the identity, precise intraciliary localization, and function of ciliary structural proteins [29,33,54,70]. However, the protein composition of some ciliary structures is still not fully resolved. Moreover, we are still far from a full understanding of how the activity of particular ciliary complexes is regulated at the molecular level, and how the coordinated activity of those complexes along the cilium length and cilium circumference translates into cilium bending and beating.

According to the generally accepted hypothesis, the signals that regulate cilia beating originate at the central apparatus [70,72]. The transient interactions between the central apparatus projections and radial spokes enable the transmission of signals to the outer doublet complexes, including the motor protein-containing dynein arms, and cilia beating regulation. These transient interactions may involve a direct CA projection–RS head contact (mechanical signals [73]) or electrostatic interactions as the cilium’s center-facing surface of the RS head is negatively charged [73,74]. However, such negatively or positively charged surfaces were not found in the case of C1a, C1b, or C1d projections. Instead, the positively and negatively charged areas were scattered on their surfaces [75]. Thus, although the replacement of 11 glutamic and aspartic acid residues, positioned at the RS head surface by serine, lysine, or alanine residues, significantly altered *Chlamydomonas* cell swimming, the significance of the putative electrostatic interactions in cilia motility requires further studies [75]. Interestingly, the chemical signals, including nucleotides produced by some central apparatus proteins (located in C1b and C2b projections), were also proposed to participate in the regulation cilia motility [75].

## 2. MCCs and Motile Cilia in Humans

Nearly all types of non-dividing human cells, at least at some stages of tissue or organ development, assemble a primary cilium, the solitary immotile sensory structure playing a key role in tissue and organ development and homeostasis [76,77,78,79,80]. In contrast, motile cilia are assembled as numerous structures (200–300 per cell) on the apical surface of epithelial MCCs. The MCCs, together with other cell types, form the epithelial lining of the upper (nasal passages, paranasal sinuses) and lower (bronchi, trachea) respiratory tracts, middle ear, Eustachian tubes, brain ventricles, efferent ductules in males, and the fallopian tube and uterus in females [81].

In a fetus, the MCCs have been transiently observed in the esophagus [82] and claimed to be transiently present in kidney tubules [83,84]. In healthy individuals with properly functioning kidneys, the epithelial cells lining the kidneys’ proximal tubules assemble solitary primary cilia. Surprisingly, under certain disease conditions, some of those epithelial cells can differentiate into MCC-like cells with a typical motile cilia 9 × 2 + 2 microtubule arrangement and at least some ciliary complexes, as can be assumed based on TEM studies [83,85,86]. However, such differentiation into MCCs is very infrequent, and scattered motile-like cilia have been detected only in biopsies obtained from individuals with tubular injuries, suggesting that it is a response to the pathological conditions [87,88].

It is also worth noting that the primary cilia assembled by the beta cells of human and mouse pancreatic islets exhibit, as has been recently described, ATP- and glucose-dependent motility, likely driven by the dynein activity. However, the mechanism generating cilium motion is not clear. TEM analyses revealed that most likely one out of nine outer doublets is shifted to the axoneme center, giving rise to an 8 × 2 axoneme with a centrally positioned doublet (8 × 2 + 2) or singlet (8 × 2 + 1). Immunolocalization studies revealed the presence of well-known motile cilia proteins within the beta cells primary cilium shaft (DNAH5, DNAI1, DNALI1, the ODA components, N-DRC subunit, DRC4, and two central apparatus proteins, SPEF2 and KIF9), although their distribution along the cilium is not uniform [89].

A specific type of motile cilia, the nodal cilia, is assembled during embryonic development (gastrula) as a single organelle per cell in the transient structure named the embryonic node. The vortex-like movement of nodal cilia is a key factor in the determination of left–right asymmetry of the main body organs [14,90]. Homologous to motile cilium but with a significantly longer structure, the flagellum, exhibiting characteristic beating, enables sperm cell motility [91,92].

The coordinated beating of motile cilia (both within the MCC and among the neighboring MCCs) empowers the transport of the secreted fluids, mucus, and particles along the surface of the ciliated cell [17,23,93,94]. Of note, it was recently discovered that proper alignment of basal bodies in MCCs, and thus planar cilia beating, is likely regulated by a unique cilium named “the hybrid cilium” [95] positioned near the cell periphery according to the cilia beating direction. The hybrid cilium has the motile cilium ultrastructure, but the basal body has numerous basal feet like a primary cilium (the basal body of a motile cilium has a single basal foot) [95].

In humans, the planar cilia beating enables the removal of mucus-trapped particles and bacteria from the respiratory tracts (mucociliary clearance) and circulation of the cerebrospinal fluid. Moreover, it is essential to collect the ovulated oocytes and support the transport of the oocyte and early embryo to the uterus [96]. In the efferent ductules, motion generated by cilia beating protects sperm cells against aggregation and supports sperm cell transport [97,98]. The improper functioning of motile cilia, significant reduction of their number, or lack of cilia leads to (i) recurring respiratory tract infections due to insufficient mucociliary clearance, (ii) frequent laterality defects (including situs inversus totalis and heterotaxy), (iii) in some cases, reduced fertility or infertility, and (iv) rarely, hydrocephalus [99,100,101]. Such ciliary defects are caused by the deleterious mutations in genes encoding structural or enzymatic ciliary proteins, or proteins regulating cilia assembly [54], and are described as primary ciliary dyskinesia (PCD), a heterogeneous and, in the vast majority of cases, recessive genetic disorder. Till now, it has been shown that mutations in nearly 60 genes can cause PCD [102]. It is generally accepted that so-far identified PCD-related genes account for approximately 70–80% of all PCD cases, while in the remaining 20–30% of cases, the genetic background is as yet unknown, calling for further studies of both samples obtained from patients during the biopsies and cilia in model organisms and cultured human MCCs.

The improper assembly or dysfunction of cilia may also have a secondary (non-genetic) origin and can be acquired due to the external conditions to which ciliated cells are exposed. Such changes are known as secondary/acquired ciliary dyskinesia and, in contrast to congenital PCD, are usually local and temporary [103,104,105].

In the next chapters, we focus on the MCCs and cilia beating-generated motion in human reproductive tracts. We summarize the old microscopic observations regarding the MCCs’ distribution in the fallopian tube, uterus, and efferent ductules and present a current understanding of how cilia defects affect female and male fertility. Because of the number of recently published splendid reviews concerning abnormalities in sperm flagella and male fertility [92,106,107,108,109], we excluded this subject from this work.

## 3. MCCs in the Female Reproductive System

Female fertility depends upon the proper functioning of the ovary, uterine tube (the fallopian tube), and uterine endometrium. The physiology of the female reproductive system is regulated by the hormonal changes during the menstrual cycle (for review [110]). The alterations in the ovary (the ovarian cycle) are coordinated with changes in the endometrium (the uterine cycle). The typical menstrual cycle consists of 28 days and is divided into a preovulatory/follicular phase (ovarian cycle) corresponding to a proliferative phase (uterine cycle, days 5–14 in the classical cycle, when day 1 is the first day of bleeding) and a postovulatory/luteal phase (ovarian cycle) corresponding to a secretory phase (uterine cycle, days 15–28) [111,112]. The hormonal changes cause, among other effects, remodeling of the epithelium lining the inner surface of the fallopian tube and the uterus, both having the same developmental origin [113,114]. Beautiful SEM images of the entire female reproductive tract can be found [115].

### 3.1. MCCs in the Fallopian Tube

#### 3.1.1. The Fallopian Tube Morphology

**Figure 2:**
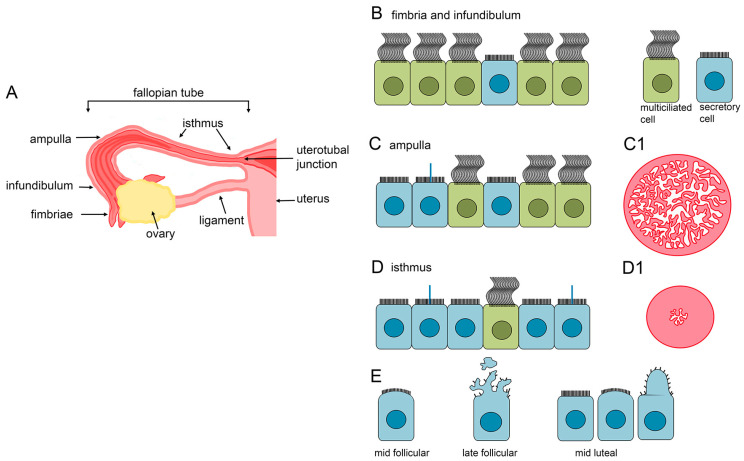
Changes in the proportion of MCCs and secretory cells along the human fallopian tube. (**A**) Schematic representation of the cut-open fallopian tube, with fimbria surrounding a part of the ovary on one end and showing the connection to the uterus on the other end; (**B**–**D**) schematic representation of the epithelium showing the proportion of the MCCs (green) and secretory cells (blue, some with a primary cilium) in (**B**) fimbria and infundibulum, (**C**) ampulla, and (**D**) isthmus; (**C1**,**D1**) a scheme of the cross-section through (**C1**) ampulla and (**D1**) isthmus showing the organization of the mucosal folds; (**E**) schematic representation of the changes of the secretory cell apical surface (based on [119,125]).

The uterine tube, also known as the oviduct or, in humans, the fallopian tube, connects the ovary to the uterus. It enables sweeping of the cumulus–oocyte complexes (COC) released during the ovulation into the tube and supports transport of the COC, migration of the sperm cells to the place of fertilization, fertilization, and, if the fertilization takes place, transport of the early embryo to the uterus. The wall of the fallopian tube is built of three layers, (i) most inner, longitudinal folds-forming mucosa, (ii) most outer, serosa, and positioned between those two, (iii) sero-muscular layer. At the macro scale, the fallopian tube can be divided into five functionally distinct regions or segments: (i) fimbria, the flattened finger-like or petal-like protrusions [116,117] forming a chalice-like structure, extending from (ii) the infundibulum and enabling pick-up of the ovulated oocyte, (iii) ampulla, where the fertilization takes place, (iv) isthmus, serving as a sperm cell reservoir, and (v) uterotubal junction, which opens to the uterine cavity (Figure 2A). The complexity of the mucosal folds projecting into the tube lumen grows from isthmus to ampulla and infundibulum (Figure 2C1,D1) [111,112,118,119,120,121,122,123], with relatively minor differences in the number of ampulla and infundibulum folds [121]. The branches and cavities formed by the mucosal folds likely affect sperm cell movement [124].

#### 3.1.2. Fallopian Tube Epithelial Cell Morphology and Structure

The inner surface of the fallopian tube is covered with monolayer columnar epithelium. Because of the changes in the cells’ height, it may appear pseudostratified, especially just before ovulation [119]. Based on histological, SEM, and TEM-based observations (see below) conducted mainly 40–50 years ago, morphologists distinguished four types of cells in the fallopian tube epithelium: (i) the secretory cells and (ii) MCCs that constitute the vast majority of the epithelial cells, (iii) small scattered cells described as peg cells or intercalated/intercalatory cells [126], and (iv) round basal/reserve cells [127], which now are recognized as intraepithelial T-lymphocytes [128].

The apical surface of the MCCs is nearly flat or slightly domed and covered with (i) numerous motile cilia that are approximately 10 µm and (ii) short microvilli [119,125,129]. The motile cilia assembled by MCCs are longer than the primary cilia formed by the secretory cells (SEM images [130,131]). The fallopian MCC cilia have a classic 9 × 2 + 2 microtubule skeleton and typical ciliary complexes. Their movement is likely generated by the activity of the same set of the dynein arm proteins as in cilia assembled by MCCs in the airways [132,133]. MCCs in the fallopian tube exhibit regular metachronal beating and generate flow in the direction of the uterus [134,135]. In vitro analyses of the fallopian tube tissues showed that such cilia activity enables the transport of the fluorescent beads along the tube surface at a mean velocity of 32 µm/s [132].

The ratio of MCCs and secretory cells changes along the fallopian tube. In fimbria, the MCCs dominate and constitute approximately 60–80% of the epithelial cell population, and their number decreases to approximately 30–40% in the isthmus (Figure 2) [119,136,137,138]. However, there is little variation in the ratio of MCCs to secretory cells during the menstrual cycle [118,119,120,126,129,137,138,139,140].

Data regarding the number of MCCs in the fallopian tubes at the end of pregnancy (obtained from patients undergoing cesarean section) are contradictory. Seki et al. reported that the MCCs in fimbria were strikingly less frequent (only ~20–30% of the epithelium cell population) and only scattered in the isthmus [141]. Moreover, within 3–5 days after the cesarean section, the number of MCCs was further reduced, and the cilia were fewer and shorter [141]. In contrast, Patek and co-authors reported that at mid-pregnancy (18–20 weeks), at the time of the cesarean section (ampulla and isthmus), and 3–5 days after delivery (infundibulum), the proportion of MCCs to secretory cells was similar to that in non-pregnant women [142].

After menopause, the number of MCCs in the fallopian tubes is reduced compared to females in the reproductive age but nearly unaltered in the case of hormone replacement therapy (HRT) [119,125,143,144,145].

The presence of MCCs and ciliogenesis were also reported in the fimbria/infundibulum region in 18–20-week-old fetuses, initially as scattered cells with 1–2 cilia and next as cells with a “crown” of growing cilia [129,140,146].

Recent analyses of the airway’s MCCs led to the identification of the hybrid cilium playing a role in the uniform orientation of all motile cilia in the MCC. The same authors found that in PCD patients with a mutated gene encoding cyclin O, the MCCs assemble one to two cilia, which are most likely the hybrid cilia [95]. Thus, one can speculate that, similarly, those one to two cilia of MCCs in a fetus’s tube are hybrid.

The number of secretory cells increases from the fimbria to the isthmus. The apical surface of the secretory cells is covered with numerous microvilli that are frequently accompanied by a solitary primary cilium [130,131,147]. Of note, although not commented on by the authors, the structures resembling primary cilia are also visible on some early SEM images [119,126,142].

Peg cells are infrequent epithelial cells. Most often, their apical part does not reach the epithelium surface [119,148] (for review [149].) Initially, it was suggested that peg cells are secretory cells that underwent secretion or apical bulge separation [119,139]. Later, because the localization of peg cells resembled that of stem-like cells (expressing CD44 and ITGA6 markers), it was suggested that some cells classified earlier as peg cells could have stem cell-like properties [148]. These data agree with recent studies [128].

The application of modern methods such as single-cell RNA sequencing to analyze several tens of thousands of cells from different parts of the fallopian tube obtained during surgery revealed that the population of the tubular epithelium cells is much more heterogeneous [128,150,151]. The sub-populations of the secretory (PAX8+) and MCCs (FOXJ1+) can be further divided into subgroups. In the case of MCCs, those groups represent (as could be expected) not-fully differentiated MCCs, differentiated MCCs, and likely senescent MCCs [128,150]. Importantly, there are also transitioning/intermediate cells expressing both secretory (KRT7) and MCCs (CAPS) markers [128,151]. This last finding agrees with observations that MCCs can differentiate from the subpopulation of PAX8-expressing cells in neonatal mouse oviducts [152].

#### 3.1.3. Cilia Beating Frequency

Data regarding cilia beat frequency (CBF) are inconsistent, likely because of differences in experimental conditions. When measured at 37 °C, CBF ranged between 6–7.3 Hz [134], “at room temperature” CBF was determined to be, on average, 5.3 Hz [153], 5–6 Hz [154], or 3.5 Hz (at 25 °C, in unspecified fallopian tube segment) [132]. Moreover, according to some researchers, cilia beat in a similar fashion independent of the tube region or phase of the menstrual cycle [155], while others observed some alterations [134,153]. According to [134,153], in the ampulla and isthmus, cilia seem to beat a little faster during the secretory phase than during the follicular phase, but only Lyons and co-authors [153] claimed that cilia in the fimbria also beat faster in the secretory phase (mean value ~5.8 Hz) compared to the proliferative phase (~4.9 Hz). On the other hand, only [134] reported that cilia in the fimbria beat slightly faster than cilia in the ampulla and isthmus but stated that their CBF does not change during the menstrual cycle, while [154] reported higher CBF in the ampulla than in the fimbria. Generally, the in vitro estimated average CBF is in range with values obtained using real-time measurements during laparoscopy or laparotomy [156]. The CBF is sensitive to hormone levels and increases when cilia are exposed to the follicular fluid [157]. In vitro studies showed that the presence of progesterone [154,158] and testosterone [159] reduced CBF, while the presence of estradiol stimulated CBF [158].

#### 3.1.4. Hormonal Regulation of the Epithelial Cells

The morphology of the apical surface of MCCs and cilia length remain basically unchanged during the menstrual cycle [118,119,126,129,139,160]. Similarly, only minor changes in MCC morphology were reported during mid-pregnancy [161], delivery, or just after delivery [142].

Detailed studies of 98 fallopian tubes obtained from women in early and late follicular or luteal phases revealed a small increase in MCCs (4–8%) during the follicular phase and ovulation. The increase in MCC number was most apparent in the fimbria [138]. These observations agree with SEM data showing ciliogenesis in the fimbria and infundibulum during the follicular phase [161]. In contrast, cells with growing cilia were not observed in the ampulla or isthmus and during the secretory phase, independent of the fallopian tube fragment [146].

Very recent studies suggest that ciliation can also be induced by changes in the viscosity of the surrounding fluid. During the follicular phase, secretory cells release different factors into the tube lumen, changing fluid viscosity, with the highest values just before ovulation. In vitro analyses of the fallopian tube epithelial cells (FTECs) under conditions mimicking in vivo changes showed that the elevated viscosity of the culture medium enhanced cell ciliation [162].

The ovarian hormones affect other processes, not only ciliogenesis. During the late follicular phase, in the fimbria, ampulla, and isthmus, the height and mitotic index of the epithelial cells were also slightly changed (of note, the difference in the mitotic index in the isthmus was not significant) [138].

The microscopic observations suggesting some increase in the number of MCCs at the follicular phase agree with the in vitro studies, showing that exposure of the primary culture of human tubal epithelium cells [163] or assembloids [164] to estradiol, whose level is high during the follicular phase, increases the ciliation and the expression of the FOXJ1 marker, respectively. Opposite to estradiol, prolonged progesterone or testosterone treatment (in transsexual women) diminished the number of MCCs. Women after prolonged (one year) progesterone therapy had approximately 10–12% fewer MCCs in the fimbria and ampulla; the effect on MCCs in the isthmus was less apparent (~3%) [138,161].

Opposite to MCCs, the shape of the apical surface of the secretory cells, the level of secretion, and subsequently, the amount of cytoplasmic debris in the tube lumen, change during the menstrual cycle [119,125].

The menstrual cycle-dependent alterations of the secretory cells are especially apparent in the isthmus but rather discrete in the fimbria. At the early follicular phase, the apical surface of the secretory cells is domed, especially in the isthmus. During the mid- and late follicular phase, the protrusions become more apparent and branched. Just before ovulation, the protrusions are remarkably prominent, reaching even above the cilia’s distal ends (tips). The fragments of protrusions likely separate from the surfaces of secretory cells, as numerous cellular particles and debris were observed in the isthmus lumen. The progressing alterations of the secretory cell surface correlate with the increase in the activity of secretory cells and the accumulation of cytoplasmic debris. The epithelial cells’ surface activity, as well as cytoplasmic debris, are no longer observed at the early/mid-luteal phase, when the apical surface of the secretory cells is again flat or slightly bulged. The cells regain limited secretory activity during the late luteal phase [119,125].

#### 3.1.5. Conditions Affecting MCCs

Several factors can affect CBF in human fallopian tubes. Adrenomedullin (ADM), a peptide hormone with an anti-inflammatory activity, is highly expressed in the tubal epithelium and, to a lower extent, in some macrophages. ADM localizes mainly in the apical part of ciliated cells and cilia, except when cells are grown under conditions that mimic the early luteal phase (a time of maximum ADM expression). Under such conditions, ADM is distributed throughout the whole epithelial cell. On a macro scale, the level of ADM in ciliated cells increases from fimbria to isthmus. In vitro, the ADM treatment enhances smooth muscle contraction and CBF in a dose-dependent manner [165,166].

In contrast, the pro-inflammatory cytokine interleukin-6 reduces CBF [167]. Recently, Wang and co-authors proposed a model connecting ADM, interleukin-6, and -8 with increasing risk of ectopic pregnancies in females with fallopian tube inflammation (salpingitis) [168]. In patients suffering from either salpingitis or tubal ectopic pregnancy, the level of ADM in the fallopian tube epithelium is significantly reduced, while the pro-inflammatory response and level of expression of implantation-associated factors (E-cadherin, active β-catenin, LIF, and HoxA-10) are enhanced, especially in samples obtained from patients with a tubal pregnancy. The authors proposed that a reduced level of ADM affects macrophages and thus contributes to the elevated expression of interleukin-6 and -8, which in turn enhance the expression of implantation-associated factors in tubal epithelial cells. In parallel, the reduced level of ADM together with the elevated interleukin level reduces CBF [168].

The presence of numerous cilia significantly increases the apical surface of the MCCs, which accommodates receptor and channel proteins. In the human fallopian tube, similar to that of mice, the TRPV4 (transient receptor potential vanilloid 4) enables the detection of tubal fluid viscosity. It localizes in the MCC apical membrane and in cilia [169]. Activation of TRPV4 by a synthetic agonist, 4αPDD, causes calcium influx and an increase of CBF in MCCs in the primary culture of hamster oviductal cells [170]. Thus, also in humans, MCCs likely sense tubal fluid viscosity (a function independent of cilia’s ability to beat) and respond by changing CBF to shift the cargo. Interestingly, in women with tubal pregnancy, the level of TRPV4 in the fallopian tube epithelium is reduced. Moreover, lower expression of TRPV4 correlates with an increasing concentration of progesterone and levonorgestrel treatment (an emergency contraceptive measure) [169].

The epithelial sodium channel ENaC is another example of a protein enriched in MCC cilia of the female reproductive tract [171]. ENaC regulates the volume and osmolarity of the extracellular fluids [172,173]. Enuka and co-authors estimated that cilia in MCCs enlarge the apical cell surface over 70 times. Considering the high number of MCCs in the fallopian tube epithelium, especially in the fimbria, and the accumulation of ENaC in cilia, it is probable that cilia can significantly contribute to the control of the periciliary fluid/uterine fluid osmolarity and volume [171].

The number and activity of MCCs in the fallopian tube can also be affected by external factors. The epithelium of fimbria, a part adjacent to the ovary, is repeatedly exposed to the damaging activity of factors present in the follicular fluid, which is released during ovulation (the follicular fluid can cause DNA damage and lipid oxidation), and thus, some cells can be damaged [174,175]. The MCCs can also be damaged by pathogens. In fallopian tube explants cultured in the presence of *Neisseria gonorrhoeae*, the bacterial cells adhere to the apical surface and invade the secretory cells but not MCCs. However, the CBF in the non-invaded MCCs adjacent to invaded secretory cells is reduced, and eventually, the MCCs are damaged and shed [176] (for review [177]).

#### 3.1.6. Role of MCCs

Our understanding of the significance of the fallopian tube MCCs is becoming increasingly complete. Recent elegant studies in mice showed that the deletion of two microRNAs, miR-34b/c and miR-449, the post-transcriptional regulators of gene expression known to play a role in ciliogenesis [178,179,180], causes infertility in female mice [179] due to defective multiciliogenesis and, in consequence, the inability to pick up the ovulated oocytes [96]. Thus, the MCCs that constitute the major fraction of fimbria epithelial cells play a crucial role in collecting the ovulated oocytes. Moreover, during ovulation, the follicular fluid released into the peritoneal cavity enters the fallopian tube [120]. In vitro, the presence of the follicular fluid significantly increases CBF (6.34 Hz versus 4.2 Hz in culture medium control) in human fallopian tube explants [157]. Thus, it is likely that there is also a temporal enhancement in vivo of cilia activity in the fimbria during ovulation.

Besides collecting the ovulated oocyte (cumulus–oocyte complex), the MCCs in the fallopian tubule likely also perform other functions. It is proposed that the coordinated beating of cilia in the uterine tube supports the muscle contraction-generated flow of the tubal fluid/secretions and together enable transport of the cumulus–oocyte complex and, next, the early embryo to the uterus. A lack of (or reduced) transport rate may end up in embryo implantation in the uterine tube (ampulla or isthmus) and in ectopic pregnancy. The early data regarding MCCs in females with ectopic pregnancy are inconsistent. The SEM-based examination of the inner surface of the fallopian tubes obtained from women after hysterectomy (control group, normal uterine tube structure) and those with ectopic pregnancy showed that the number and distribution of MCCs cells, as well as the number and length of cilia, are similar in both groups [118], while other researchers [181] observed significantly fewer ciliated cells in biopsies obtained from patients with tubal pregnancy.

In miR-34b/c and miR-449-deficient mice, early embryo transport was possible but less efficient than in wild-type animals [96]. These observations agree with the long-standing opinion that the coordinated beating of MCC cilia supports uterine tube fluid flow and the movement of the oocyte generated by muscle contractions.

MCCs may also contribute to the movement of sperm cells from the uterus to the isthmus and, next, the ampulla. For fertilization to occur, sperm cells deposited in the vagina have to (i) travel through the uterus and a part of the fallopian tube and (ii) undergo physiological changes called capacitation, a process that requires direct contact between the tubular epithelium and the sperm cell [182,183,184,185,186,187,188]. In vitro observations of the interactions between sperm cells and the fallopian tube explants suggest that MCCs are likely involved in this process. First, in the presence of sperm cells, the CBF increases, suggesting some contribution of the cilia-generated movement of tubal fluid to sperm cell migration [189]. Consistent with this, the migration of sperm cells in the female reproductive tract of miR-34b/c and miR-449-deficient mice is slower [96]. However, if tubal fluid flow indeed supports sperm cell migration, what is the mechanism? The coordinated beating of cilia propels the fallopian tube luminal fluid toward the uterus, and thus the flow direction is opposite to that of the sperm cell swimming. It turns out that the current generated by cilia provides the directional clue guiding the sperm cells (positive rheotaxis) first to the isthmus and, next, to the ampulla [190,191,192].

It has also been reported that in the isthmus, MCCs participate in sperm cell binding [193], although to a similar extent as secretory cells [194]. However, the motile cilia are assembled as numerous organelles per cell, and thus the surface accessible for interaction with sperm cells is significantly increased. Moreover, densely packed, erected cilia protruding above the cell surface can effectively entrap sperm cells. It is postulated that such storage of sperm cells and their gradual release from the epithelium surface decrease the possibility of polyspermic fertilization [188]. Although the primary cilium is considered a sensory structure, the motile cilia can also perform such functions [8,9,10,11]. Thus, one could speculate that attachment of the sperm cells to cilia or factor(s) released by sperm cells could also initiate intraciliary signaling. Finally, the immotile or aberrantly beating cilia can cause local accumulation of damaging factors and debris, leading to pathological processes including tumorigenesis [175,195].

Recent significant progress in the in vitro culture of human fallopian tube cells, both as organoids [196,197] and assembloids [164], will enable a better understanding of the molecular mechanisms behind morphological and physiological changes in the epithelial cells, including the significance of MCCs in normal and pathological processes.

### 3.2. MCCs in the Endometrium of the Uterus

#### 3.2.1. Uterus Anatomy

**Figure 3:**
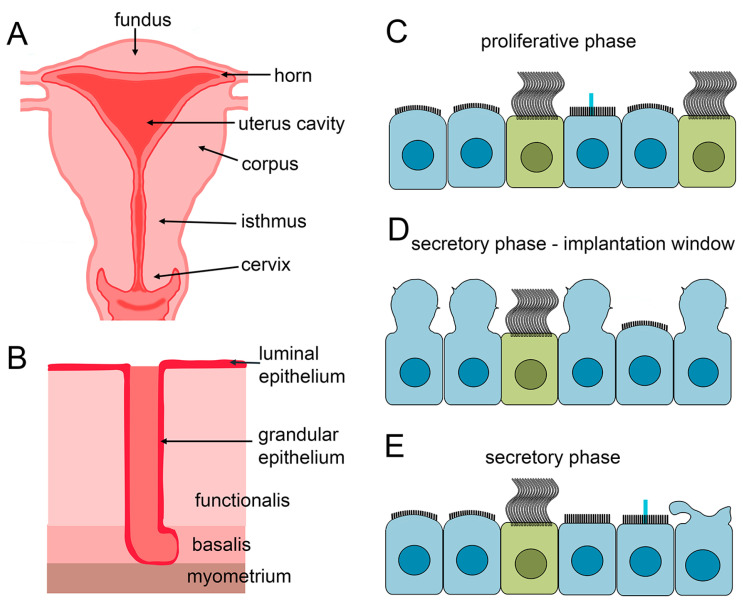
Changes in the proportion of MCCs and secretory cells in the epithelium lining the inner surface of the uterus. Schematic representations of (**A**) uterus; (**B**) uterine gland; (**C**–**E**) luminal epithelium, showing the proportion of the MCCs (green) and secretory cells (blue, some with a primary cilium) during (**C**) proliferative phase, (**D**) implantation window, and (**E**) late secretory phase. Note flat or slightly dome-shaped secretory cells with numerous slender microvilli during the proliferative phase (**C**), secretory cells with a single, large, bulb-like protrusion, a pinopode, during the implantation window (**D**), and a secretory cell with a regressing pinopode in a secretory phase (**E**). Based on the SEM and TEM images [204,205,206,207].

The uterus, a pear-shaped female reproductive organ, is the site of embryo implantation and fetus development. Anatomically, the uterus is subdivided into five regions: (i) uterine cornua or horns, where the uterine tubes connect to the uterus, (ii) fundus, the part above the entry of the fallopian tubes, (iii) corpus or body, which is the main part of the uterus and the place of blastocyst implantation, (iv) isthmus, the neck-like region below the corpus, and (v) cervix, connecting the uterus with the vagina (Figure 3A). Histologically, the uterus comprises three tissue layers: the outer layer named perimetrium, the smooth muscle-containing middle layer, myometrium, and the endometrium, the inner layer lining the uterine cavity. The endometrium has two histologically and functionally distinct layers: the superficial functional part (superficial stratum functionalis), which undergoes hormone-dependent monthly cycles of proliferation, differentiation, and removal (menstruation), and a basal part (stratum basalis) that is not shed during the menstruation [198,199] and houses numerous types of stem/progenitor cells, enabling regeneration of the functional layer (for review [200,201,202,203]).

The functional layer of the endometrium comprises a single layer of columnar epithelial cells and underlying stroma (for the stroma cell composition, see [208]). Depending on the location, the uterine epithelium is named luminal or glandular (Figure 3B). The luminal epithelium faces the uterine cavity, while the glandular epithelium forms glands that delve into the stroma, up to the myometrium [209,210]. Some genes are differently expressed in cells forming these epithelia. The luminal epithelial cells have a higher expression of COX1 and KRT5, while cells forming the glandular epithelium have an elevated expression of SCGB2A2 marker [211].

#### 3.2.2. Cellular Composition of the Uterine Epithelium and Changes During the Menstrual Cycle

Similar to the fallopian tube, the uterine epithelium is composed of non-ciliated secretory cells and MCCs [212]. However, in contrast to the fallopian tube epithelium, (i) in the uterus, the MCCs are far fewer, (ii) in some parts of the uterus, the number of MCCs is invariant, while in others, changes during the menstrual cycle, (iii) the apical surface of the non-ciliated cells changes dramatically during the implantation window, forming pinopodes (see below), and (iv) the epithelium, as a part of the functional layer, is shed and regenerated in monthly cycles.

The uterine MCCs were described more than one hundred years ago in German-language reports mentioned by [213,214]. In both the proliferative phase (corresponding to the ovarian follicular phase) and the secretory phase (corresponding to the ovarian luteal phase), the morphology of MCCs, basically does not change. Their apical surface is rather flat. Besides 50–60 motile cilia (data from the cornua [139] and corpus [215]) of estimated length 4–6 µm (cornua) [139] and 8 µm (corpus) [215], the MCCs form short microvilli [136,215,216]. The TEM ultrastructural data of the endometrial MCCs are limited. Most of the motile cilia present in the uterus have a typical 9 × 2 + 2 microtubule arrangement [206,216,217,218]. A beautiful image shown in figure 6 of [216] shows numerous 9 × 2 + 2 cilia, all with unidirectional oriented central apparatuses, suggesting directional cilia beating [219]. On the other hand, Pearson-Farr and co-authors suggest that in isolated glands, the central pair orientation is random, or the central apparatus is missing. These observations led the authors to the conclusion that cilia likely beat asynchronously [218].

Interestingly, some atypical cilia were also observed in the uterus. Based on cilia cross-sections, the anomalies included lack or duplication of the central pair (with or without the displacement of one of doublets to the cilium center), cilia with more than one axoneme (giant cilia), intracytoplasmic cilia, and others [206,216,217,218]. According to [217], the atypical cilia were identified in biopsy samples from 10 out of 15 women, independent of the phase of the menstrual cycle. Thus, atypical cilia may be formed in healthy individuals. On the other hand, as the secretory phase progresses, the number of MCCs substantially diminishes (please see below for details), likely due to their resorption or disassembly. Thus, it is possible that some atypical cilia are, in fact, resorbed or degraded cilia. For example, it is implausible that MCC forms atypical intracytoplasmic axonemes. However, it was reported that in some cell types, the motile cilia are removed by their internalization [220,221]. Thus, it is possible that the intracytoplasmic axonemes are axonemes of the internalized cilia that will be degraded within the cell. If such an assumption is correct, that would be an interesting phenomenon.

If intracytoplasmic axonemes represent resorbed cilia, the other images of atypical cilia obtained from the same samples (swollen cilia, cilia with broken microtubules) could also represent stages of cilia degradation [222]. For example, four central microtubules could arise as an effect of breaking and shifting the broken microtubules into the cilium shaft, forming a fragment without central microtubules and fragments with four microtubules. Similarly, giant (double-axoneme) cilia could be formed by breaking and shifting the fragment of the same axoneme. Such aberrations were observed in some ciliary mutants in unicellular model organisms [223]. Alternatively, under some conditions, the ciliary membrane of adjacent cilia could fuse, forming multi-axonemal structures, as can be seen in some PCD biopsies [224] or after viral infection [225]. To summarize, the ultrastructural alterations observed at least in some atypical cilia could be an effect of secondary defects due to cilia resorption.

In contrast to MCCs, the apical surface of the secretory cells undergoes extreme remodeling (Figure 3C–E), changing from flat or slightly dome-shaped, with numerous slender microvilli, during the early and mid-proliferative phase to a bulging surface, with fewer and shorter microvilli at the late proliferative phase [112,136,226]. As the cycle progresses, the apical surface of secretory cells forms a single, large, bulb-like protrusion called a pinopode or uterodome, initially having some short microvilli but becoming smooth when fully developed. Pinopodes are transient, progesterone level-dependent structures, reaching above the cilia tips, formed only during the so-called implantation window (days 19–21, a period of maximal receptivity) [205,206,207,227,228,229,230,231,232]. On average, pinopodes are formed by ~88% of the non-ciliated cells, while the remaining ~12% maintain microvilli. At the same time, the MCCs constitute 7% of the entire cell population of the luminal epithelium [231].

During the proliferative phase, the estrogen stimulates the restoration of the functional layer shed during menstruation and its initial thickening. It also induces the expression of the progesterone receptor. During the early secretory phase, the endometrium further changes, preparing for the implantation. The morphological and physiological changes in most of the uterine area are accompanied by changes in the proportion between MCCs and secretory cells, with a general tendency for the number of MCCs to increase in the proliferative phase and decrease during the secretory phase (see below for details). This agrees with the recent finding showing that during the proliferating phase, there are not only ciliated cells but also pre-ciliated cells (CCNO+) [211].

After successful implantation, the endometrial surface undergoes significant reorganization, transforming into the so-called decidua (for review [233,234]). In the first weeks of gestation, the MCCs are still observed, but their number gradually decreases as pregnancy progresses [235]. By the eighth week, only abnormally shaped cilia can be occasionally found in the fundus [236].

If the fertilization does not occur, the endometrium functional layer is shed (menstruation) and the newly forming functional layer originates from the stem cells present in the basal layer [200,201,202,203]. In the regenerating endometrium, ciliogenesis can be detected on the seventh to eighth days of the menstrual cycle [237,238].

In the uterine epithelium, the ratio of MCCs to secretory cells changes not only during the menstrual cycle but also with aging [239,240]. Comparative in silico transcriptomic studies of endometrial transcriptomes (23–49 years old women with regular menstrual cycle) from the Gene Expression Omnibus [239] and the comparison of the transcriptomes from endometrial biopsies obtained from women of different ages (20–27 and 47–50 years old) all undergoing HRT (on day 5 of progesterone administration) [240] revealed an up-regulation of a significant number of genes related to cilia assembly and function in older women. These findings correlate with the lower frequency of successful implantations in older women.

#### 3.2.3. MCCs in the Different Regions of the Uterus

Most early scanning and transmission electron microscopy-based studies provided characteristics of the uterine epithelium only during a certain phase of the menstrual cycle, within a certain uterine region, or without giving details about the studied area. Therefore, we summarize only those studies that provided details regarding the analyzed uterus part and phase of the menstrual cycle when samples were obtained.

The transition between the fallopian tube and the uterus (the utero-tubal junction) is marked by a significant decrease in the number of MCCs [139]. In the fallopian tube fragment adjacent to the transitional area, the MCCs constitute approximately 10% of the epithelial cell population, and their number and cell morphology are similar during the proliferative and secretory phases. In contrast, the secretory cells exhibit some alterations in the apical surface due to secretory activity during the proliferative but not secretory phase, similar to that observed in other parts of the fallopian tube. In the cornua of the uterus, the MCCs constitute only approximately 5% [139] or even only ~1% [136] of the epithelial cell population, but, similarly to the tube, their number is similar during the monthly cycle. The MCCs are slightly more numerous around the gland openings. In contrast to MCCs, the morphology of the apical surface of the secretory cells changes during the cycle (indicating their secretory activity), from domed and microvilli-rich during the proliferative phase to protrusion-forming during the secretory phase and “collapsed, emptied” protrusions during the late secretory phase [136,139,236].

In the fundus, the MCCs are also infrequent and generally visible as solitary cells surrounded by secretory cells. Their number slightly increases near the gland openings [136,227,236]. During the early secretory phase, the MCCs constitute ~4–15% of the epithelial cells, but their number varies, even between areas from the same biopsy sample [227]. At the same time, the secretory cells form prominent, bulge-like protrusions. Regretfully, as far as we know, it has not been determined whether the MCC number in the fundus changes during the different phases of the menstrual cycle.

The corpus of the uterus is the most frequently studied part. The MCCs are present in both luminal and glandular epithelia [213,226,237,241], separately or in groups [136,226], and are slightly more frequent in the vicinity of the gland opening [136,204,226,237,242]. The number of MCCs fluctuates during the menstrual cycle. During the early proliferating phase, the MCCs constitute only ~2.5–3.5% of the luminal and glandular epithelium. Under the influence of estrogen, the number of MCCs increases reaching the maximum (~6%) in the early secretory phase [136,213,226], for early data review, see [112]. In contrast, according to [241] the number of MCCs during the proliferating phase is much higher, reaching even 20% of the cell population. Starting with the mid-secretory phase, the number of MCCs substantially diminishes to ~1–2% in the late secretory phase, suggesting cilia resorption [226].

An increase in the number of MCCs was observed in the isthmus, a part of the uterus near the cervix. The MCCs were distributed separately or in groups and accumulated around the gland openings [213,216,236]. Most likely, their number does not vary significantly during the menstrual cycle [213,236], while the structure of cilia changes from normal in the late proliferative phase to drooped in the early secretory and blistered and rough in the late secretory [236]. The epithelium lining the inner part of the cervix (endocervix) is composed mainly of non-ciliated cells, while MCCs are very rare [213,242].

#### 3.2.4. In Vitro Organoid and Assembloid Studies

Recently, the fluctuation in the number of endometrial MCCs during the menstrual cycle was confirmed by immunolocalization studies [243]. In organoids representing the glandular epithelium (GLS) exposed to estrogen E2 (resembling a follicular phase), the MCCs constitute nearly 10% of the epithelial cell population [243] or even 30% [211,244], which is more than what was reported in early SEM studies. Strikingly, in parallel studied assembloids, composed of both epithelial and stroma cells and exposed to the hormones to resemble phases of the menstrual cycle, MCCs constitute 5% of the cell population under the proliferation phase conditions and 1.3% under the conditions resembling the secretory phase [244]. These data are in good agreement with the summarized early SEM studies. Moreover, while in GLS organoids, the cilia length does not change, in endometrium samples obtained during the secretory phase and in epithelial and stroma cell assembloids grown under the secretory phase conditions, cilia become slightly shorter [244]. The transcriptomic studies revealed that 1288 cilia-related genes are down-regulated in MCCs present in assembloids compared to MCCs in GLS organoids. Together, the scRNA-Seq data and hormone-response experiments led authors to the conclusion that the endometrial stromal cells may induce the reduction of the number of ciliated cells and regulate their dynamics during the menstrual cycle [244].

The differentiation of MCCs is strictly controlled and requires the inactivation and activation of several factors [93]. Early in vitro observations showed that estrogen, which is elevated during the proliferative phase, stimulates both, cell proliferation and ciliogenesis [214]. Transcriptomic analyses of the glandular epithelium organoids revealed that estrogen E2 affects the expression of multiple genes, causing either their down- or upregulation. Among the upregulated genes were the *PGR* gene, encoding the progesterone receptor, genes encoding transcription factors, and ciliary proteins performing structural and regulatory functions [243].

More detailed analyses of this organoid revealed that the estrogen E2 was sufficient to induce differentiation of approximately 9% of organoid cells into MCCs. Strikingly, the number of MCCs increased nearly ten times when, simultaneously with estrogen treatment, the NOTCH signaling pathway was inhibited, probably due to NOTCH1 receptor downregulation [243]. Importantly, the inhibition of NOTCH alone was insufficient to trigger ciliogenesis, leading authors to the conclusion that estrogen E2 is necessary and sufficient for MCC differentiation, while NOTCH inhibition significantly potentiates this process [243].

#### 3.2.5. Significance of MCCs

The role of endometrial multiciliated cells in normal physiological processes and in endometrium pathology is far from being understood. Comparative analyses of MCC cilia in endometrial glands obtained by endometrium biopsies from control, healthy women and women with a history of recurring pregnancy loss or with subfertility revealed more ultrastructural alterations and reduced CBF in females with fertility problems compared to healthy ones [218], suggesting that MCCs and cilia beating play an important role in this organ. Some researchers proposed that the flow generated by cilia could spread secretions produced in glands [115,235,241]. Others suggested that cilia could support the movement of the sperm cells [226].

It has also been proposed that cilia could play a role in sensing and signaling. Endometrial cilia are approximately 6–8 µm long, erect cell organelles protruding above the endometrium surface and thus, similar to the primary cilia, could perform sensory functions and participate in the regulation of the volume of the uterine fluid and possibly in sensing uterine fluid osmolarity [171].

Single-cell transcriptomic analyses of the human endometrial cells obtained during biopsies and derived organoids have enabled temporal and spatial mapping of the gene expression under normal and pathological conditions, as well as characterization of the subpopulations of cells based on the expression of the specific marker genes [211,245,246,247,248]. The gained data brought significant progress in our understanding of the processes taking place in endometrial cells and the mechanisms governing their differentiation. However, we are still far from a full understanding of the significance of MCCs and motile cilia in the endometrium physiology.

### 3.3. Cilia and Female Infertility

Recurrent upper and lower respiratory tract infections are the main reason why individuals affected by PCD seek medical advice. Not surprisingly, the diagnostic tests and a vast majority of the PCD-related research concentrate on the MCCs from airways. At the same time, data concerning fertility are limited for numerous reasons, including the patients’ young age, personal patient decisions, and clinician approach. For example, in one of the studied PCD cohorts, out of 167 adults, the fertility data were available for only 80 individuals. In the case of the remaining patients, the data were not available, or patients did not try to conceive [249]. Based on the analyses of PCD cases with available information regarding patient infertility (defined as prolonged problems with conception), it was estimated that approximately 60% of females with PCD had reduced fertility or were infertile [106,249,250]. Interestingly, six out of twenty-two infertile women gave birth to a child with the help of assisted reproductive technology (ART) [249] suggesting that defects in the fallopian tube cilia, rather than in endometrium cilia, could cause infertility in those patients. Unfortunately, there is no information on whether the remaining 16 infertile patients tried ART.

More than 50 genes have been identified as PCD-causative genes [251]. The severity of PCD symptoms depends upon the mutated gene, the type of mutation, and the effect of the mutated or eliminated protein on cilia assembly and functioning. Although the number of women with PCD and fertility problems included in the above-mentioned studies was low, the outcomes of those analyses were similar: mutations in several genes invariably coincided with female infertility. These were (i) *CCDC39* and *CCDC40,* encoding proteins forming the so-called “molecular ruler” that marks the 96-nm ciliary unit and defines the place where N-DRC, IDAs, and RS are docked [67], (ii) genes encoding axonemal dynein assembly factors, required for IDA and ODA preassembly [252], and (iii) *HIDIN*, a large protein forming C2b projection of the central apparatus [253,254]. On the other hand, mutations in genes encoding radial spoke proteins or dynein arms including the dynein heavy chain *DNAH5*, known to cause severe respiratory problems, did not inevitably cause subfertility/infertility (for details please see tables in [106,249,250]).

In the fallopian tube MCCs, the localization of DNAH5 and some other ciliary proteins was similar to that observed in the airway’s MCCs [132]. Single-cell transcriptomic studies of human and murine nasal, bronchial, oviductal, and ependymal epithelia showed that genes encoding cilia-related proteins were expressed at similar levels [255]. Thus, one could assume that in individuals with PCD, cilia of MCCs from different locations share similar ultrastructural and motility defects. However, the clearance of mucus with entrapped inhaled particles out of the airways depends solely upon the motile cilia activity. In contrast, the transport of the oocyte and early embryo in the fallopian tube also depends upon the contraction of the uterine tube muscles and is only supported by the cilia movement. On the other hand, the cilia beating frequency is reduced as the density of the surrounding environment increases [256,257]. Thus, different extracellular conditions in the airways and in reproductive tracts may differently affect the motion of cilia with the same ultrastructural defect.

In mice, there are no mechanisms that substitute for cilia motion in picking up the ovulated oocyte. However, some oocytes are collected in humans with defective cilia as the spontaneous conception occurs in PCD-affected females. Thus, if other mechanisms do not exist in humans, perhaps even reduced or aberrant cilia motion is sufficient to collect some of the oocytes, and only a lack of cilia or their complete immotility precludes this process.

Generally, defects in the fallopian tube and endometrial cilia in PCD-affected females are poorly documented. We found one report documenting endometrial cilia ultrastructure in a female with PCD, showing a lack of the central pair in 87% of cilia [258]. Without a doubt, in all cohort studies, the number of participants was too low to draw a definitive conclusion, but the observed tendencies between mutated genes and fertility are well-matched. More detailed analyses of cilia in the reproductive tract are needed to better understand the role of motile cilia in the fallopian tube and endometrium in healthy female physiology and cases of sub- and infertility.

## 4. MCCs and Cilia in Efferent Ducts of the Male Reproductive System

### 4.1. Efferent Ductules Morphology

A comprehensive review of the role of motile cilia in the efferent ductules and male fertility in animal models was recently published [259]. To avoid repetitions, here, we will focus on data regarding the efferent ducts and MCCs in humans, and we will only briefly mention the key findings in the animal models when needed.

**Figure 4:**
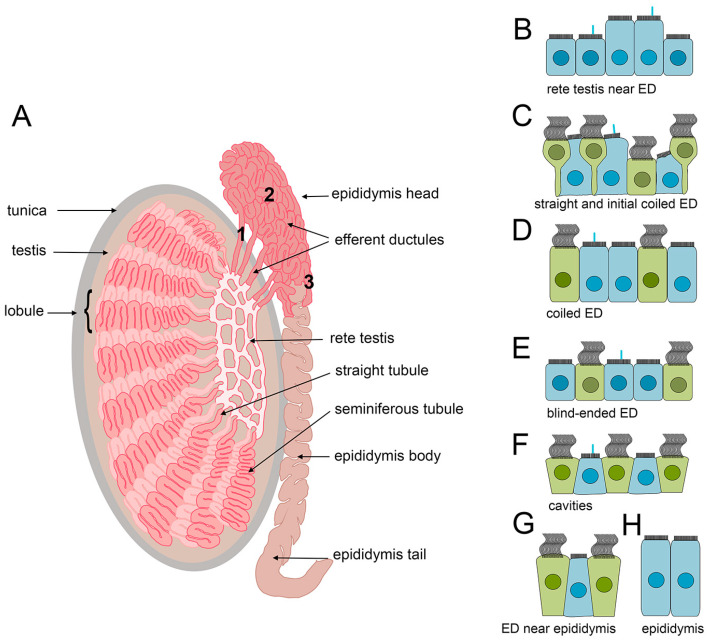
Schematic representation of the epithelium composition in efferent ductules. (**A**). Scheme of the testis and epididymis; (**B**–**H**) schematic representation of the monolayer epithelia lining the luminal surface of the indicated parts of the reproductive tract (1–3 in image 4A) based on TEM images [264]; (**1**) straight tubules and initial part of the coiling fragment of the efferent ductules, image 4C; (**2**) efferent ductules, images 4D–G; (**3**) epididymis, image 4H; the reabsorptive cells (blue) with microvilli and primary cilia, MCCs (green) with numerous motile cilia (please note the changes in cell shape and height); (**B**) irregular epithelium composed of non-ciliated cells in rete testis, near the efferent ductule junction; (**C**) irregular epithelium composed of MCCs and reabsorptive cells in the straight tubule and initial part of the coiling fragment of the efferent ductules; (**D**) typical columnar epithelium in coiled parts of the efferent ductules; (**E**) cuboidal epithelium in blind-ended efferent ductules; (**F**) cuboidal epithelium in efferent ductules’ cavities; (**G**) columnar epithelium in efferent ductules, close to the junction with the epididymis; (**H**) columnar epithelium in epididymis composed of non-ciliated cells. ED: efferent ductules.

The efferent ductules, also named efferent ducts, ductuli efferentes, or vasa efferentia, originate from the mesonephric tubules [260] and constitute the largest part of the epididymis head (caput epididymis). They link the rete testis, the network of tubes and cavities continuous with seminiferous tubules [261,262] to the epididymis tubule (Figure 4). Efferent ductules are formed as numerous structures, initially reported as five to seven [263] or eleven to fifteen tubules [264,265]. Recent three-dimensional reconstruction of the region between the rete testis and epididymis revealed that approximately 23 tubules lined with efferent ductule-specific epithelium exit the rete testis. Some of those tubules terminate after several millimeters. The remaining tubules (13–17) proceed toward the epididymis, forming long, proper efferent ductules. Most ductules fuse one by one to form the tubule that joins the end of the epididymal duct, while 4–6 ductules connect independently, laterally to the epididymis tubule [266]. The total length of all efferent ductules in analyzed samples ranged from approximately 2 to 4 m [266]. The efferent ductules leaving the rete testis are almost straight but become extensively coiled and form densely packed lobules in the epididymis head [263,264,265,266].

### 4.2. The Morphology of the Efferent Ductules Epithelial Cells

The inner (luminal) surface of the efferent ductules is lined with the monolayer epithelium, which differs in its morphology from the epithelia in rete testis and epididymis tubules. In rete testis epithelium is cuboidal, in the efferent ductules, mostly columnar, and in the epididymis, columnar but composed of much taller cells. The change from one type of epithelium to another is abrupt, and therefore, it is relatively easy to identify boundaries between the rete testis, efferent ductules, and epididymis [263,264,266]. Moreover, in contrast to other parts of the male reproductive tract, in the efferent ducts, the epithelium besides microvilli-forming cells (further called “reabsorptive”) also contains MCCs, whose role in the efferent ducts was only recently addressed in a mouse model [97].

The efferent ductules are lined with columnar epithelium, but there are some differences along the ductules’ length. In the short, straight fragments originating from the rete testis and in adjacent coiling fragments, the epithelium consists of cells of variable height, both ciliated and non-ciliated (pseudostratified epithelium), and thus is called “irregular” (Figure 4C). Moreover, the MCCs have a wineglass or reverse bottle-like shape, with the thinner part at the lamina propria. In the coiled efferent duct fragments, the epithelium is columnar, with flask-shaped MCCs. In the cavities and blind-ended ductules, the epithelium is cuboidal, with the MCCs having a more regular shape in the blind-ended tubules and a more trapezoid shape in the cavities (Figure 4E,F). Additionally, some differences in reabsorptive cells’ and MCCs’ shapes, number, and size of the vacuoles, granules, and mitochondria were observed in different fragments of the efferent ductules [264]. It remains to be discovered if this variability suggests some functional differences or simply reflects the physiological states of the same cell types. The identity of efferent duct cells was recently addressed using modern techniques [267,268].

The reabsorptive cells and MCCs constitute approximately 61% and 36% of the epithelial cell population. The remaining 3% are macrophages and T-lymphocytes (also observed earlier [269]), present at the base or within the epithelium. Most reabsorptive cells assemble a single cilium, likely a primary cilium. The ultrastructure and function of the primary cilium have not yet been addressed in human efferent ductules. The length of the primary cilium varies from 0.2 to 15 µm (on average 2.2 µm) [270]. Cilia assembled by MCCs are significantly longer than primary cilia, ranging from 5.6 to 16.4 µm (on average 9.5 µm [270]). They are also longer than motile cilia assembled by MCCs in the airways [271].

Based on immunofluorescence studies, it seems that there are two types of MCCs in the efferent ductules. Co-labeling of the human efferent ductules sections with anti-acetylated tubulin antibodies (a cilia marker) and anti-cytokeratin-5 (epithelial intermediate filament) antibodies revealed that MCCs (acetylated-tubulin positive cells) can be either cytokeratin-5 positive or negative [272]. Regretfully, the single-cell RNA sequencing analyses did not shed more light on the putative diversity of MCCs in the efferent ductules [268,273]. Moreover, as the authors pointed out, the cluster of ciliated cells could contain sperm cell contamination [268]. A re-analysis of the transcription profiles of ciliated cells based on *DNAH5* expression level would enable the elimination of such contaminations, as dynein heavy chains encoded by *DNAH5* are not present in sperm cells (instead, sperm cells have dynein heavy chains encoded by *DNAH8* and *DNAH17*) [274].

### 4.3. Role of MCCs in the Efferent Ductules

We can only speculate about the role of MCCs and motile cilia in the human efferent ductules based on data obtained in animal models. As observed in mice, the beating of motile cilia in efferent ducts is uncoordinated, as the direction and speed of cilia beating, as well as the power stroke direction, can vary between neighboring MCCs [97]. These observations suggest that in efferent ducts, the luminal fluid and sperm cells are mixed by the motile cilia movement, rather than shifted along the epithelium surface. In mice, lack of efferent ductules cilia due to the elimination of miR-34b/c and miR-449a/b/c clusters caused, among others, sperm cells’ clumping. Based on those observations, the authors postulated that MCCs generate mixed currents that protect sperm cells from forming aggregates that could block the ductular lumen [97].

### 4.4. Ciliary Gene Mutations and Male Infertility

In humans, MCCs with mutations in the *CCNO* or *MCIDAS* genes (encoded proteins are involved in basal bodies multiplication [17]), assemble only a few cilia [275,276], and cause a PCD sub-type called RGMC (reduced generation of motile cilia). Till now, only twelve male patients have been diagnosed with a *CCNO* mutation, and seven of them are 5–15-year-old children [275,277,278]. Out of five adult patients, one was reported to be a father to a child, while no data was provided for the other four [275]. Mutations in *MCIDAS* have been identified so far in four male patients [276,279], but only in the case of one patient, who was diagnosed with azoospermia, were the morphology and ultrastructure of efferent ducts analyzed [279]. As expected, the efferent duct epithelium was nearly deprived of cilia, and the ductule lumen was dilated and filled with sperm cells [279], a phenotype typical for a loss of cilia function in efferent ducts.

Mutations in *DNAH5* are long-known causes of PCD, with severe respiratory problems due to significantly reduced cilia motility [280]. Since, in the male reproductive system, *DNAH5* is expressed exclusively in the efferent ductules, if infertility occurs, it is caused by defects in motile cilia but not in sperm flagella. Male patients with PCD caused by *DNAH5* suffer from azoospermia and oligozoospermia, but there was also a case of a patient with a normal sperm number [98]. Moreover, patients with PCD caused by a *DNAH5* mutation can be fertile [108,249,250], suggesting that perhaps aberrant or reduced cilia motion in efferent ductules can be sufficient to protect some sperm cells from aggregation. It is striking that while individuals with *DNAH5* mutation have severe respiratory symptoms, they can still be fertile. Perhaps the answer is in the mode of cilia beating. In the airways, cilia beating is coordinated within the MCC and between MCCs, whereas in efferent ductules, MCC beating is uncoordinated [97].

We are only beginning to understand the significance of MCCs in the efferent ducts and the role of motile cilia activity in male fertility. Most of the data have been obtained in animal models, mostly mice with eliminated miRNAs (miR34/449), transcription factors (Mcidas, Gmnc/Gemc1, E2f4/5), or proteins involved in the differentiation of multiciliated cells (CcnO) and motile cilia assembly and/or functioning (Dnah5) (for review [259]). Males of all these mutants shared similar phenotypic alterations: dilatation of the seminiferous tubules, rete testis, and efferent ductule lumen; agglutinations of sperm cells in efferent ductules; and infertility [97,98,281,282]. Although one can assume that mutations in these genes, or other genes involved in multiciliogenesis, cause efferent duct insufficiency and, in consequence, infertility, in most cases, there are no ultrastructural or functional data supporting this theory.

## Data Availability

Not applicable.
